# Development and validation of an interpretable machine learning model and online web-based calculator based on social-ecosystem theory for early prediction of postpartum depression: a longitudinal study

**DOI:** 10.3389/fpubh.2025.1685305

**Published:** 2025-10-17

**Authors:** Shusen Lin, Peng Wang, Chongyu Yue, Guofang Kuang, Xuefei Han, Yanxia Zhang, Xiaojing Wang, Min Wang, Shanshan Huan, Xinwei Zhang, Ping Tan, Huawei Li

**Affiliations:** ^1^School of Nursing, Qingdao University, Qingdao, Shandong, China; ^2^Qingdao Municipal Hospital, Qingdao, Shandong, China; ^3^Affiliated Hospital of Qingdao University, Qingdao, Shandong, China

**Keywords:** postpartum depression, machine learning, clustering algorithms, predictive learning models, risk assessment

## Abstract

**Background:**

Postpartum depression (PPD) has emerged as a global public health issue that can cause significant harm to mothers and their families. Currently, there is an urgent need for a robust early risk prediction model to enable accurate predictions of postpartum depression in hospitals.

**Methods:**

This was a longitudinal study. Using social ecosystem theory, we collected multi-dimensional and multi-angle risk factors for early postpartum depression from delivery to discharge, and conducted 42-day postpartum follow-ups using the Edinburgh Postnatal Depression Scale (EPDS). We strictly adhered to the Transparent Reporting of a Multivariable Prediction Model for Individual Prognosis or Diagnosis (TRIPOD) checklist, used 10 machine learning (ML) algorithms to construct and validate the prediction model, and employed the Shapley additive explanation (SHAP) algorithm to explain the model. Risk stratification was performed through K-Means clustering analysis, ultimately resulting in an clinical screening tool for early PPD risk prediction.

**Results:**

The results showed that by comparing the performance of prediction models constructed by the 10 ML algorithms, the model constructed using the random forest algorithm was selected as the best, with an area under the receiver operating characteristic curve (AUC) of 0.91 (95% CI: 0.85–0.96) and 0.77 (95% CI: 0.70–0.85) in internal and external validation. Low risk probability (0, 0.26], medium risk probability (0.26, 0.63), and high risk probability (0.63, 1) were obtained through K-Means clustering analysis, and the SHAP value of the model was interpreted. Finally, we developed an online risk prediction calculator.

**Conclusion:**

This study developed an interpretable risk prediction model for early PPD, which may help healthcare providers to identify and implement intervention measures early, preventing the occurrence of PPD.

## Introduction

Postpartum depression (PPD) is a mental disorder characterized by core symptoms such as low mood, loss of interest or pleasure, and diminished energy. This condition is frequently accompanied by psychological symptoms and somatic manifestations (1). PPD is the most prevalent psychological complication following childbirth and remains a significant research focus worldwide. It typically occurs 2–4 weeks after childbirth (2). Many risk factors are associated with its development, and the symptoms can even persist for up to 1 year after childbirth (3, 4). Research indicates that 80% of women experience a form of postpartum “blues” after childbirth, while 15% develop severe perinatal depression (5).

PPD has emerged as a global public health concern (6). Women with PPD frequently have hostility, self-neglect, lower tolerance to external stimuli, as well as poor responsiveness to their infants’ needs (7, 8). It not only disrupts the mother-infant relationship and reduces breastfeeding rates (9), but also negatively impacts children’s cognitive, behavioral, emotional, and physical development (8, 10). In severe cases, it may even result in suicidal or infanticidal behaviors (11). Research indicates a global prevalence of PPD reaching up to 14% (12), with significantly higher rates observed in developing countries, particularly China, where prevalence reaches 21.4%. Furthermore, within the 24-week postpartum screening window, the prevalence of PPD may rise from 12.9 to 17.4%, demonstrating an increasing trend (12, 13).

In 2019, the U.S. Preventive Services Task Force (USPSTF) updated guidelines to recommend counseling interventions for high-risk individuals with perinatal depression, contingent on depression screening implementation (14). There remains an urgent need for accurate early screening tools for PPD. However, the time points of the influencing factors collected by previous studies covered more data after discharge, which made it difficult to achieve accurate risk prediction of early PPD in hospitals. Moreover, most of them had no theoretical support (15, 16). Furthermore, existing models were rarely translated into clinically usable visual formats for healthcare providers (17). Therefore, this study was conducted to address these limitations.

With the rapid development of machine learning (ML) algorithms, artificial intelligence (AI) has gradually been extended to the research field of PPD, aiming to help medical staff identify high-risk mothers early. In the current field of nursing research, the use of risk prediction models for risk identification is a hot topic and challenge. These models mainly include two types. One constructs non-parametric prediction models through ML algorithms in data mining, while the other constructs parametric prediction models through traditional statistical methods, such as Logistic regression and linear regression models. When these models are visualized, they can help medical staff make clinical decisions and implement early interventions. ML algorithms can be classified into Supervised Learning, Unsupervised Learning, Semi-supervised Learning, and Reinforcement Learning. The most commonly used method is supervised learning, which is a subcategory of ML and AI. It is defined as using a labeled dataset to train an algorithm in order to classify data or accurately predict an outcome (18, 19). Compared to risk prediction models constructed using traditional statistical methods, those built with ML algorithms offer unique advantages. Many scholars have developed risk prediction models using algorithms such as decision trees, random forests (RFs), support vector machines (SVMs), naive Bayes, and other ML-supervised learning algorithms to identify PPD early, with good performance. Model visualization through nomograms has yielded positive results in clinical practice, but some shortcomings remain, which prevent early identification and convenient application in hospitals (17).

American scholar Charles H. Zastrow proposed the Society Ecosystem Theory based on previous studies, dividing the social ecological environment into three levels: micro, meso, and macro systems. This theory emphasizes the mutual influence and interaction between individuals and the social environment in which they live. It describes and analyzes the relationship between individuals and their environment, offering a comprehensive and systematic perspective for researchers. In addition, the theory also emphasizes the importance of considering the integrity of individuals when addressing research problems, advocating for analysis and intervention from multiple systems and perspectives (20). Due to changes in postpartum identity and an increase in responsibilities (21), the causes of PPD become more complex. Therefore, it is of great significance to apply this theory to collect and analyze early risk factors for PPD from multiple dimensions and perspectives. This approach can also provide reliable support for the construction of prediction models in the later stages.

This study was a longitudinal study. The primary objective was to construct a risk prediction model for PPD at 42 days postpartum using data collected from delivery to hospital discharge with ML algorithms. Because identifying PPD becomes substantially more difficult after maternal discharge, and symptoms peak at 4–6 weeks, it is critical to use these data for early identification and intervention of PPD within the hospital.

This study aimed to develop and validate early risk prediction models for PPD using 10 classical ML algorithms, including Logistic Regression, Decision Tree, Random Forest (RF), Support Vector Machine (SVM), eXtreme Gradient Boosting (XGBoost), K-Nearest Neighbor (KNN), Naive Bayes, Adaptive Boosting (AdaBoost), Light Gradient Boosting Machine (LightGBM), and Bayesian Network. Shapley Additive Explanation (SHAP) values were used for model interpretation and risk stratification. Finally, an online web-based risk calculator was developed for accurate early prediction in the hospital setting. This study might promote the application of interpretable and practical risk prediction models in early PPD prediction and provide a reliable screening tool for the development of subsequent risk management strategies.

## Methods

### Participants

This study was approved by the Ethics Committee of Qingdao Medical College of Qingdao University (QDU-HEC-2021114). All participants provided informed consent before entering the study. The inclusion criteria were women who had normal deliveries, with informed consent, voluntary participation in the investigation, and the ability to cooperate. The exclusion criteria included women with a history of mental illness such as depression or mental or intellectual disabilities, or those whose families had experienced major trauma or significant changes from the first 6 months of pregnancy to delivery. The samples for the training set were used for model development and internal validation, including women who delivered in the obstetrics department of our hospital between July 2023 and October 2024 and met the inclusion and exclusion criteria. The samples for the test set were used for external validation, including women who gave birth in the obstetrics department of our hospital between November 2024 and January 2025 and met the inclusion and exclusion criteria.

### Data sources

This study adopted a convenience sampling method to recruit postpartum women from the Department of Obstetrics at two leading tertiary hospitals in Qingdao, China: the Affiliated Hospital of Qingdao University and Qingdao Municipal Hospital. These institutions are classified as Grade A in China’s three-tier six-class hospital system and serve as comprehensive medical centers integrating clinical services, medical education, scientific research, disease prevention, healthcare maintenance, and rehabilitation services.

The sample size for the training set was determined using the Kendall sample size estimation method, which recommended a sample size of 5–10 times the number of the independent variables (22). There were 53 candidate independent variables in this study. Considering a 10% invalid response rate for the questionnaires, the sample size was calculated to be at least 294 mothers. The internal validation group was randomly split at a ratio of 7:3, with the internal validation group accounting for 30% of the total sample.

The test set was verified by time period, and the sample size for the external validation group was typically 1/4 to 1/2 of the sample size for the modeling group (23, 24). Taking into account a 10% invalid response rate for the questionnaires, the sample size for the external validation group was calculated to be at least 82 mothers.

### Candidate features and outcome

The Early Risk Factors for PPD Questionnaire (see Supplementary Information for details) was developed based on a literature review and the social-ecological systems theory, aiming to identify early risk factors for PPD across four levels: individual, family and interpersonal, community and hospital, and societal. The questionnaire was developed through expert panel discussions and is completed by women between childbirth and hospital discharge. It incorporates the Social Support Rating Scale (SSRS).

To evaluate maternal social support within family and interpersonal factors, the SSRS, compiled by Xiao Shuiyuan (25), was used. The SSRS consists of 10 items divided into three dimensions: subjective support (items 1, 3, 4, 5), objective material support (items 2, 6, 7), and the utilization of support (items 8, 9, 10). The total score range for this scale is 13 to 62. Scores greater than 45 indicate high social support, scores between 33 and 45 indicate moderate social support, and scores between 13 and 33 indicate low social support.

Maternal depression was followed up via telephone at 4–6 weeks postpartum, and depression was diagnosed using the Edinburgh Postnatal Depression Scale (EPDS). The EPDS was initially developed by Cox et al. (26) at Livingston and Edinburgh Health Center for assessing postpartum depression, with a reliability coefficient of 0.87 (26). The scale includes 10 items that assess emotions and psychological feelings, such as mood, fun, self-blame, anxiety, and insomnia. Each item is rated on a 4-point scale based on the severity of symptoms, with a total score range of 0–30. For this study, a score of EPDS≥10 was used as the cutoff for diagnosing depression.

### The process of data collection

Before the study began, participants were informed about the purpose, significance, and process of the study. They were given the right to voluntarily participate or withdraw from the study at any time. Data were collected using a combination of questionnaire surveys and electronic medical record inquiries. Links to the questionnaires were sent to the participants, or they were invited to scan a QR code to complete the questionnaires themselves. In some cases, paper questionnaires were provided to participants.

The Early PPD Risk Factors Questionnaire was completed from childbirth to hospital discharge, while the Edinburgh Postnatal Depression Scale was filled out via electronic questionnaires at 4–6 weeks postpartum.

### Data analysis

#### Statistical analysis and feature selection

After the questionnaires were collected, the data were entered into Excel and analyzed using SPSS 29.0 statistical software. Countable data were presented as frequency and percentage, with comparisons between groups made using the Chi-square test or Fisher’s exact probability method. Measurement data with a symmetrical distribution were presented as the mean ± standard deviation (SD), and comparisons were performed using the independent samples t-test. Data with a skewed distribution were presented as the median and interquartile range, and the Mann–Whitney U test (non-parametric) was used for comparisons. A *p*-value of < 0.05 was considered statistically significant. Variables found to be significant in the univariate analysis were included in the binary logistic regression model for multivariate analysis. The significant variables from the logistic regression analysis were then retained in the final model. Samples with short response times, identical responses to all items, or obvious outliers were considered invalid and were excluded from the analysis.

#### Model development and evaluation

In this study, Python 3.12 was used to construct a risk prediction model using 10 ML algorithms: Logistic Regression, Decision Tree, RF, SVM, XGboost, KNN, Naive Bayes, AdaBoost, LightGBM, and Bayesian Network. The training set was divided into a modeling group and an internal validation group in a 7:3 ratio using random splitting. The best model parameters were selected through grid search, and 10-fold cross-validation was used during parameter adjustment. Finally, the model was tested using both internal and external validation data, and the indicators such as accuracy, precision, recall, F1 score, area under the receiver operating characteristic curve (AUC), and 95% confidence intervals (CI) were calculated. The development process strictly adhered to the Transparent Reporting of a Multivariable Prediction Model for Individual Prognosis or Diagnosis (TRIPOD) checklist.

### Risk stratification and development of an online web calculator

To stratify the risk based on the prediction model, K-Means clustering (a fast clustering method) was used to divide the prediction probabilities from the best model into different risk levels. The subjects were divided into three groups: low, medium, and high risk.

For the K-Means clustering, three cluster centers were set, with a maximum of 10 iterations. Statistical descriptions, analysis of variance, and multiple comparisons were performed for the stratified data.

Finally, the model was visualized and an online web calculator was developed using Streamlit, an open-source Python framework for rapidly building and deploying interactive, data-driven web applications.

## Results

### Baseline characteristics

**Figure 1:**
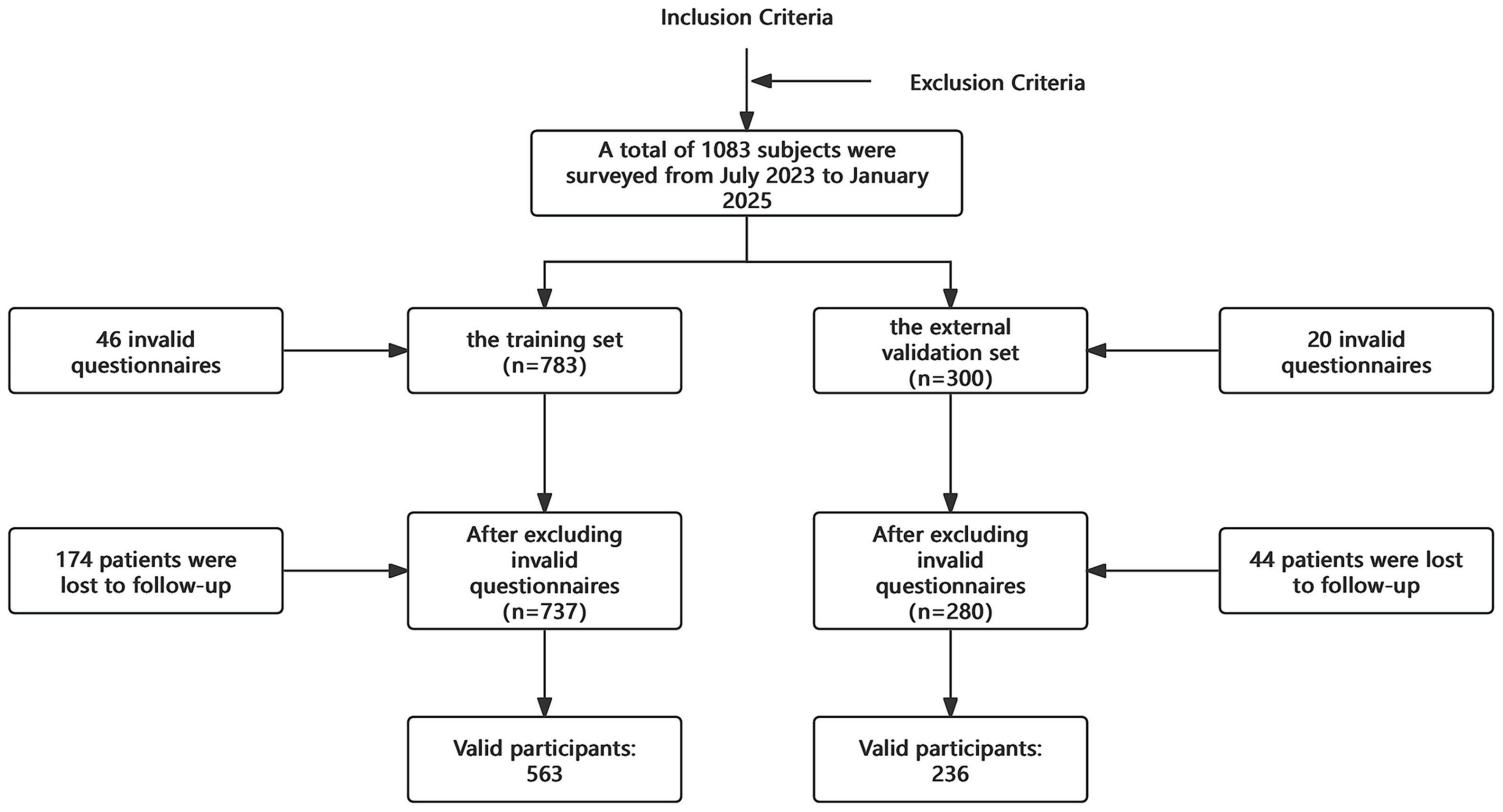
Data collection process.

A total of 1,083 pregnant women were selected between July 2023 and January 2025, with 799 valid questionnaires ultimately collected (Figure 1). Among these, 563 women were in the training set, with an incidence of PDD of 36%. In the external validation set, which included 236 subjects, the incidence of PDD was 30%. The overall incidence of PDD in the entire sample of 799 women was 34%. A descriptive of the training set data are provided in the Appendix.

### Variable screening

This study included 53 potential risk factors associated with PDD. A univariate analysis of these factors using data from the training set (see Appendix Table S1 for univariate analysis) identified 15 risk factors that were significantly associated with PDD: X8, X13, X14, X15, X20, X21, X22, X23, X37, X38.6, X43, X46, X47.2, X49.1, and X53. Subsequently, multivariate analysis using binary logistic regression and stepwise regression (step-forward conditional) (Table 1) was performed, which resulted in the identification of 7 independent risk factors (see Appendix Table S2 for the assignment of independent variables): (1) X8: Work Stress During Maternity Leave; (2) X13: Can the Husband Provide Sufficient Care? (3) X14: Postpartum Recovery Place; (4) X22: Relationship with Parents-in-law; (5) X37: Did Pregnancy Weight Gain Cause Distress? (6) X43: Current Maternal Sleep Condition; (7) X53: Level of Social Support. Collinearity diagnostic analysis of these 7 independent risk factors (see Appendix Table S3 in the for collinearity diagnosis) showed no significant collinearity (Variance Inflation Factor [VIF] < 5).

**Table 1:** Training set maternal binary logistic regression (step-forward conditional) (*n* = 563).

Variables	β-value	Standard error	*p*-value	OR	95%CI
Constant	−7.640	0.719	<0.001	<0.001	
X8-Work Stress During Maternity Leave	0.579	0.230	0.012	1.784	1.137–2.799
X14-Postpartum Recovery Place			0.019		
Reference-own home					
Postpartum care center	0.318	0.404	0.432	1.374	0.622–3.034
Others	1.130	0.410	0.006	3.095	1.387–6.908
X22-Relationship with Parents-in-law	0.992	0.167	<0.001	2.698	1.945–3.741
X37-Did Pregnancy Weight Gain Cause Distress?	2.418	0.253	<0.001	11.220	6.831–18.427
X43-Current Maternal Sleep Condition	0.659	0.100	<0.001	1.932	1.589–2.349
X53-Level of Social Support	0.578	0.175	<0.001	1.782	1.264–2.511

### Model performance

We used ten ML algorithms to construct risk prediction models and compared their performance to select the best model for developing an online web calculator to compute the early risk probability of PPD. The performance and parameters of each model’s internal and external validation are shown in Table 2.

**Table 2:** Internal and external validation evaluation indicators of 10 machine learning algorithms.

Algorithm	Accuracy	Precision	Recall	F1 value	AUC (95%CI)
Internal validation					
Random forest	0.81	0.81	0.81	0.81	0.91(0.85-0.96)
XGBoost	0.79	0.80	0.79	0.79	0.90 (0.85–0.96)
AdaBoost	0.80	0.81	0.80	0.81	0.88 (0.82–0.93)
LightGBM	0.79	0.80	0.79	0.80	0.88 (0.82–0.92)
Decision Tree	0.80	0.80	0.80	0.80	0.87 (0.81–0.92)
SVM	0.77	0.79	0.77	0.77	0.86 (0.78–0.92)
Logistic Regression	0.79	0.79	0.79	0.79	0.86 (0.80–0.91)
Naive Bayes	0.77	0.78	0.77	0.77	0.84 (0.78–0.89)
KNN	0.77	0.79	0.77	0.77	0.77 (0.70–0.84)
Bayesian Network	0.72	0.63	0.44	0.52	0.65 (0.58–0.72)
External validation					
Random Forest	0.77	0.76	0.77	0.76	0.77 (0.70–0.85)
XGBoost	0.76	0.75	0.76	0.75	0.76 (0.69–0.82)
AdaBoost	0.73	0.72	0.73	0.72	0.74 (0.66–0.81)
LightGBM	0.75	0.74	0.75	0.74	0.74 (0.65–0.81)
Decision Tree	0.74	0.72	0.74	0.72	0.73 (0.66–0.80)
SVM	0.74	0.73	0.74	0.73	0.75 (0.69–0.82)
Logistic Regression	0.74	0.74	0.74	0.74	0.75 (0.68–0.81)
Naive Bayes	0.76	0.76	0.76	0.76	0.85 (0.82–0.88)
KNN	0.76	0.75	0.76	0.75	0.69 (0.63–0.75)
Bayesian Network	0.77	0.83	0.28	0.42	0.63 (0.57–0.69)

**Figure 2:**
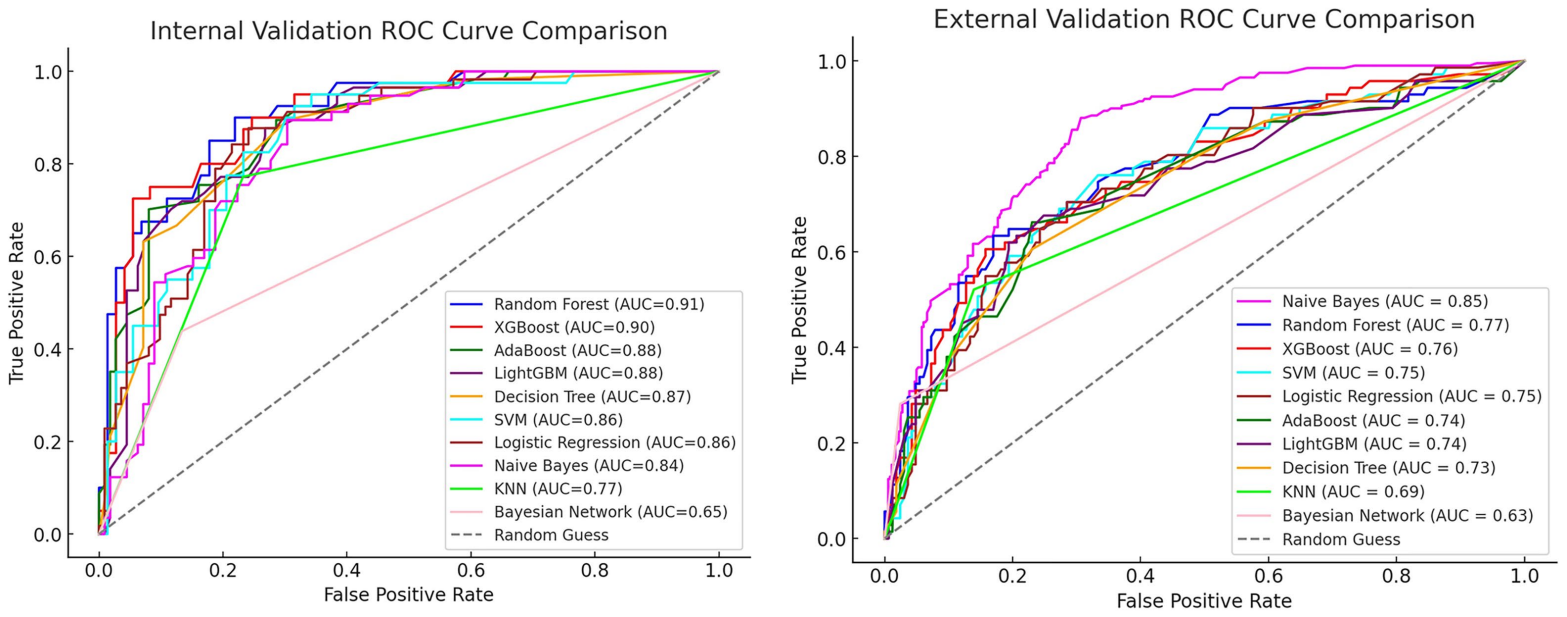
Comparison of ROC curves for internal and external validation.

After comparing the evaluation metrics for both internal and external validation, we identified the RF model as the best performing model. Its internal validation AUC was 0.91 (95% CI: 0.85–0.96), and the external validation AUC was 0.77 (95% CI: 0.70–0.85). Although the external validation result of the RF model was slightly worse than that of the Naive Bayes model (AUC = 0.85, 95% CI: 0.82–0.88), considering the overall performance in terms of internal validation, accuracy, precision, recall, F1 score, and AUC value, the RF model was finally selected as the best model. The ROC curves for internal and external validation of each model are compared in Figure 2.

### Risk stratification

We used the K-Means clustering (Fast Clustering) analysis method to stratify the risk based on the predicted probabilities from the RF prediction model training set. The participants were categorized into three risk levels: low, medium, and high risk. K-Means clustering was set to 3 clusters with a maximum of 10 iterations.

**Figure 3:**
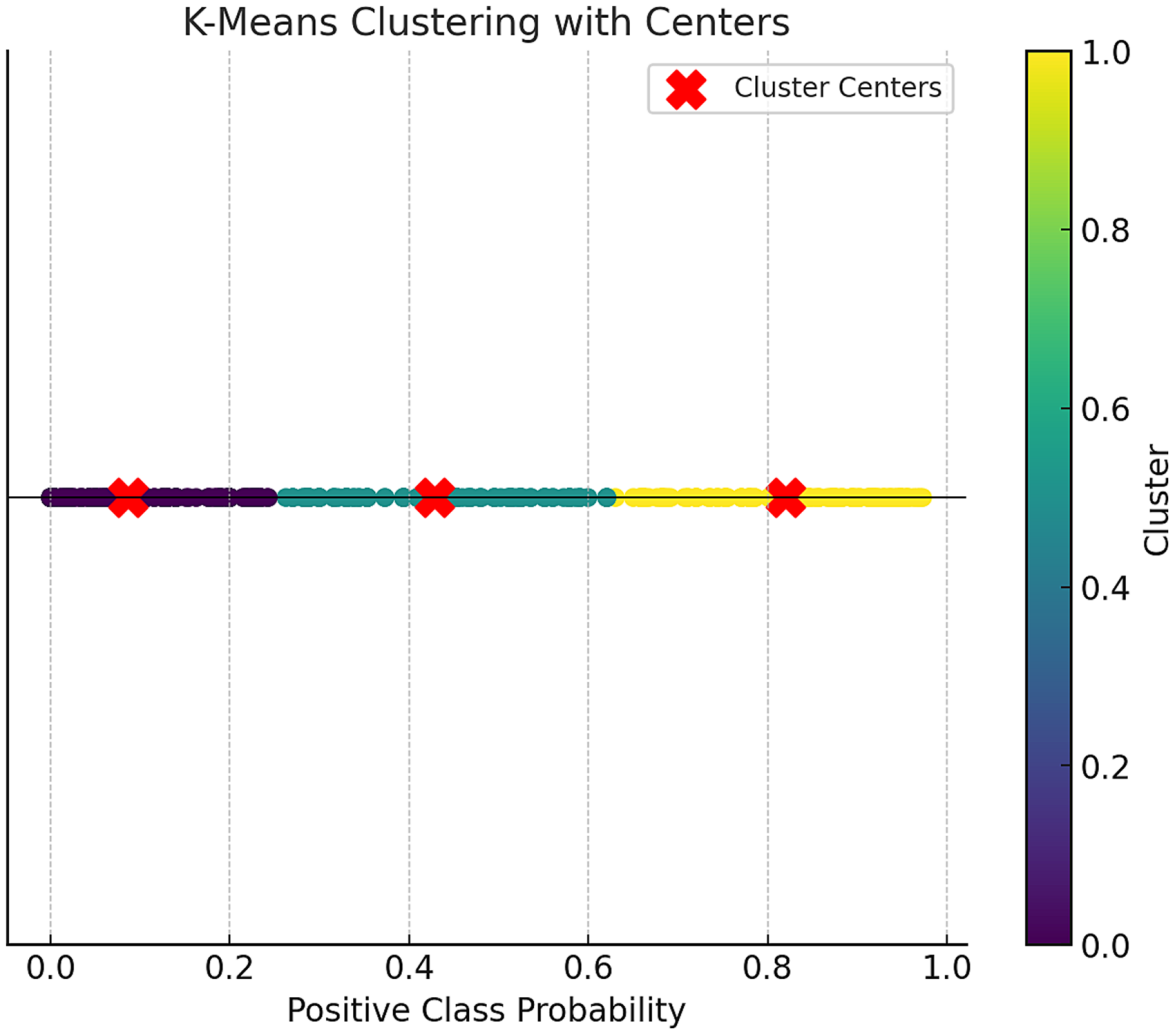
Scatter plot of K-Means clustering.

Figure 3 shows the scatter plot based on K-Means clustering, where each point represents a data point, and the points are color-coded according to their clustering labels. The x-axis represents the “Positive Class Probability” values, and the clustering labels are distinguished by color. The red “X” markers indicate the center of each cluster. Table 3 presents the statistical analysis results after the fast clustering stratification. Based on these results, the early risk probability for PPD was classified as follows: low risk (0, 0.26), medium risk (0.26, 0.63), and high risk (0.63, 1).

**Table 3:** Results of rapid cluster hierarchical statistical analysis of risk probabilities for early postpartum depression.

Cluster	Sample size	Mean	Standard deviation	Standard error	95%CI	Minimum	Maximum
1	260	0.08635	0.08072	0.00501	0.07649–0.09620	0.00000	0.24283
2	163	0.42785	0.10061	0.00788	0.41228–0.44341	0.26250	0.62043
3	140	0.81965	0.09915	0.00838	0.80308–0.83621	0.63021	0.97283

**Figure 4:**
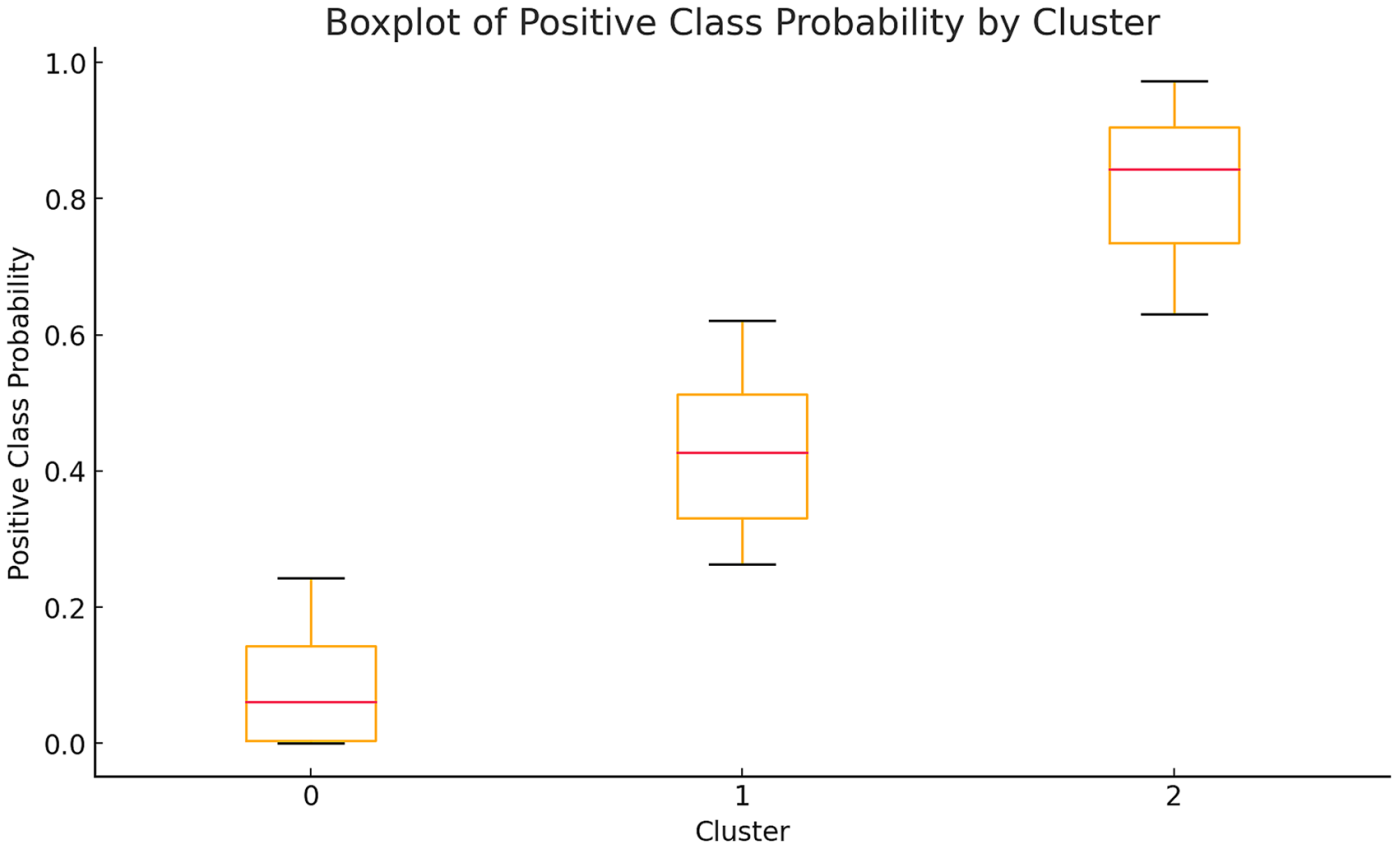
Box plots of three groups of probability distributions.

Table 4 presents the one-way analysis of variance (ANOVA) for the low, medium, and high-risk groups, showing statistically significant results (*p* < 0.05), indicating significant differences in the means between the three groups. We then conducted a least significant difference (LSD) multiple comparison (see Table 5), which revealed significant differences between each pair of groups. Finally, box plots (Figure 4) were used to display the distribution of the three groups.

**Table 4:** Post-cluster one-way ANOVA of risk probabilities for early postpartum depression.

Variable	Homogeneity of variance test		Welch’s test	
	Statistic	Significance	Statistic	Significance
Three groups of data after clustering	7.549	<0.001	2952.109	<0.001

**Table 5:** LSD multiple comparisons of the total probability mean of the three groups after clustering.

Category (I)	Category (J)	Mean difference (I-J)	Standard Error	Significance	95%CI	
					Lower bound	Upper bound
Low-risk group	Medium-risk group	−0.34150	0.00915	<0.001	−0.35947	−0.32353
	High-risk group	−0.73330	0.00960	<0.001	−0.75215	−0.71445
Medium-risk group	Low-risk group	0.34150	0.00915	<0.001	0.32353	0.35946
	High-risk group	−0.39180	0.01055	<0.001	−0.41252	−0.37107
High-risk group	Low-risk group	0.73330	0.00960	<0.001	0.71445	0.75215
	Medium-risk group	0.39180	0.01055	<0.001	0.37108	0.41252

### Model interpretation and online app

**Figure 5:**
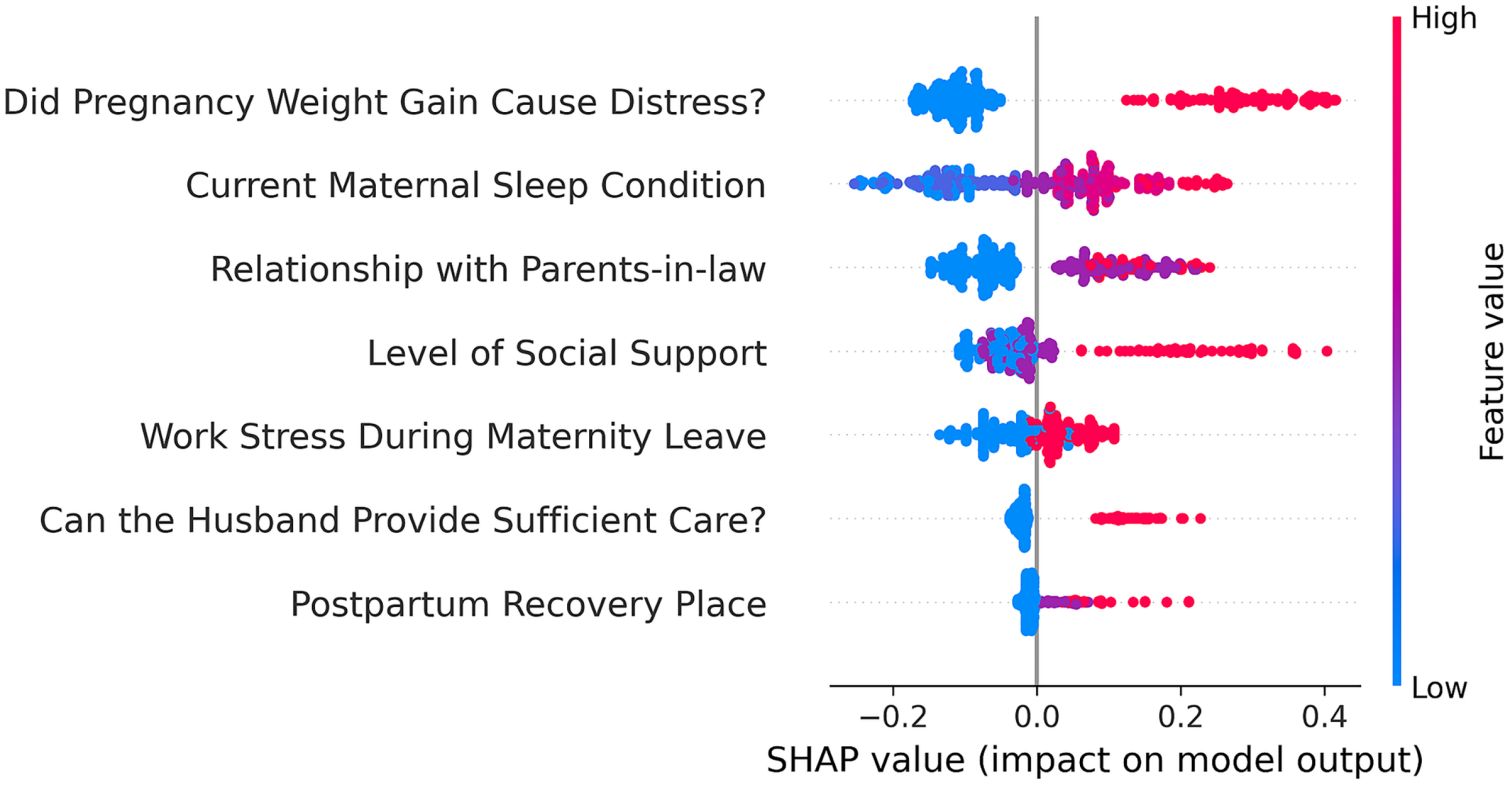
SHAP values for Random Forest.

**Figure 6:**
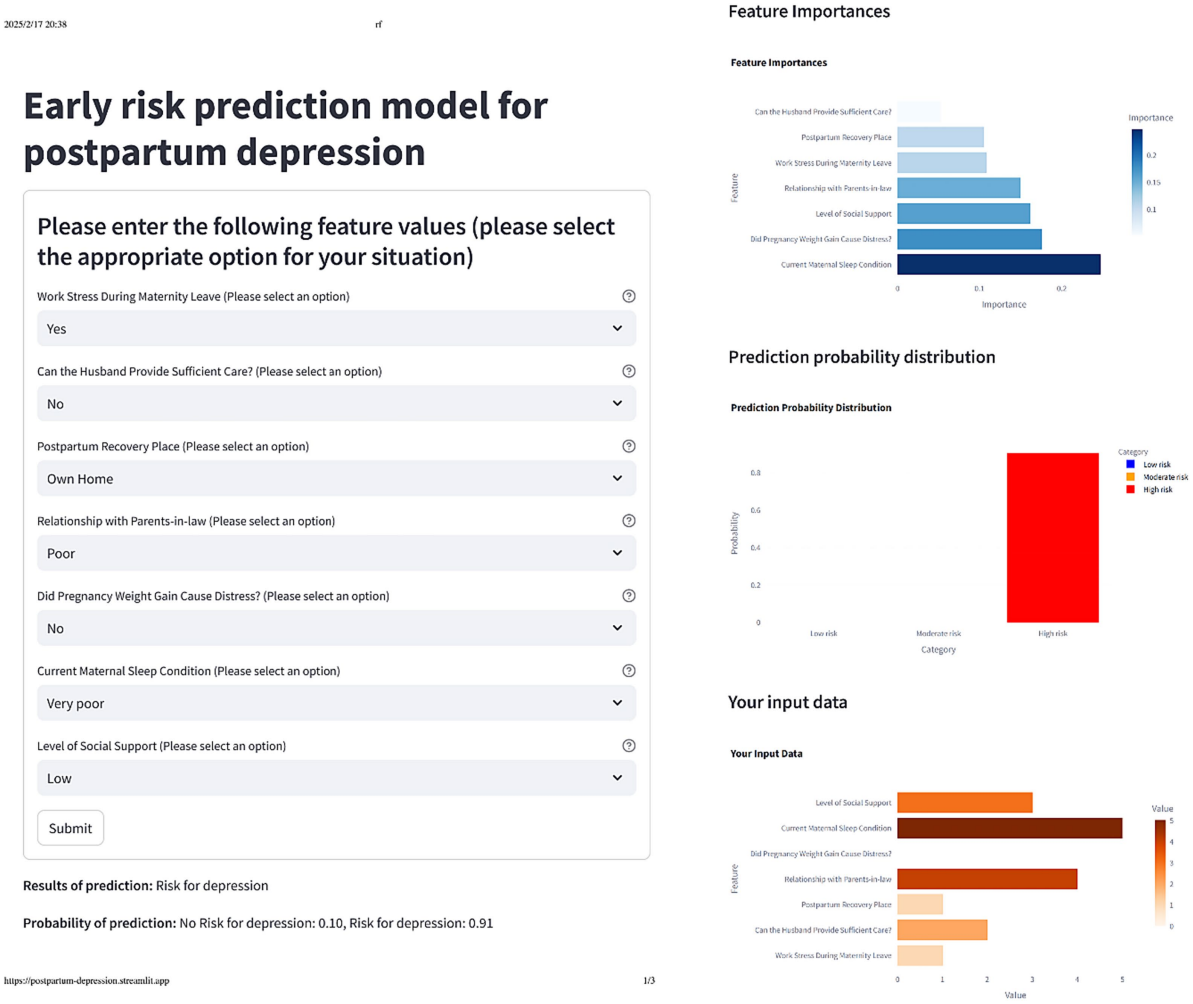
The actual display of the online web calculator after selecting the corresponding feature option.

To better understand the model’s internal workings, we used a SHAP Summary Plot to display the model’s local interpretability. Figure 5 shows the SHAP values generated from the RF model, To improve the accessibility of the model in this study, we visualized it as an online web calculator. The early risk probability of PPD can be accurately determined by inputting the options for the predictor variables in the table. The predicted probability distribution, characteristic distribution, and risk distribution are shown in detail in Figure 6 (accessible at: https://postpartum-depression.streamlit.app/).

## Discussion

Based on the social-ecological systems theory, this study explores the risk factors of early PPD from multiple dimensions and perspectives. The results identified seven independent risk factors for early PPD: (1) Work Stress During Maternity Leave; (2) Can the Husband Provide Sufficient Care? (3) Postpartum Recovery Place; (4) Relationship with Parents-in-law; (5) Did Pregnancy Weight Gain Cause Distress? (6) Current Maternal Sleep Condition; and (7) Level of Social Support. The detection rate of PPD in this study was 34%, which was consistent with the incidence reported in a meta-analysis study (27). In addition, we used ten ML algorithms to construct risk prediction models. The results showed that the model constructed using the RF algorithm demonstrated excellent performance in both internal and external validation, with AUC values of 0.91 (95% CI, 0.85–0.96) and 0.77 (95% CI, 0.70–0.85), respectively. These results indicate that the model has strong discriminatory power and clinical application value. In addition, based on the good performance of the RF algorithm, we conducted an interpretability analysis and developed an online web calculator. We recommend that users use it for early prediction of PPD.

The results of this study indicated that women with PPD were more likely to experience work-related stress in the early postpartum period compared to those without PPD. Work stress during maternity leave was identified as an independent risk factor for early prediction of PPD, which was consistent with the findings of previous studies (28). In addition, the study found that the level of social support and the sleep conditions of postpartum women were positively correlated with the occurrence of PPD, which was consistent with earlier research (29, 30).

A meta-analysis (31) also supported the idea that longer postpartum sleep duration was associated with reduced fatigue and fewer PPD symptoms. Other studies (32) have confirmed that subjective sleep scores are highly correlated with the severity of depressive symptoms. Research (33–36) suggests that insufficient sleep might be associated with an increased risk of depression, coronary heart disease, diabetes, and obesity. In addition, several studies have further explored the role of social support (37) and found that partner support is one of the most influential predictors of PPD symptoms. Friend support has also been shown to correlate with PPD symptoms, and the lack of partner support can increase the stress of raising children alone (38). These findings were consistent with the results of our study.

The results of this study also revealed that a worse relationship between the mother-in-law and daughter-in-law was associated with a higher probability of PPD, which was consistent with findings from other studies in China (39, 40). In China, postpartum women commonly cohabit with their parents-in-law, who often assist in caring for both the postpartum woman and the newborn. One of the most frequent sources of family conflict arises between postpartum women and their mothers-in-law, particularly regarding differences in caregiving approaches and expectations. Furthermore, the study found that the probability of PPD was lower in parturient women who had postpartum recuperation at their own homes compared to those recuperating elsewhere. This finding aligned with several other studies (41, 42). This may be attributed to differences in living habits, parenting styles, and dietary patterns among the cohabitants. When postpartum women recuperate in their own homes, they are more familiar with their environment and care methods, which may facilitate a smoother adaptation and contribute to better mental health.

This study revealed a positive association between maternal distress caused by gestational weight gain and PPD. During the study, some pregnant women did not experience anxiety or distress related to weight changes (either gain or loss), while others, despite not undergoing significant weight changes, were more troubled by the fluctuations in their weight (43, 44). This distress could potentially become an influencing factor for PPD. The findings of Mariam and Srinivasan also showed that 44% of mothers with prenatal psychological distress would go on to develop PPD (45). These results emphasize the importance of providing appropriate guidance during pregnancy to help women understand and accept the weight change during pregnancy and to help them develop reasonable diet and exercise intervention programs.

Many scholars have conducted research on the construction of risk prediction models for PPD. Yang et al. constructed a risk prediction model based on logistic regression and confirmed its strong predictive performance (46). Hochman et al. utilized the XGBoost ML algorithm to construct a model for classifying the risk of PPD, which showed promising results (47). To compare the effectiveness of different ML algorithms, we used 10 ML algorithms (selected according to the characteristics of the data) to construct prediction models. By comprehensively evaluating the performance of these models, we selected the most effective one. Zhang et al. also constructed a PPD risk prediction model through a variety of ML algorithms, addressing the limitations of relying on a single algorithm (48). While their model performed well, it lacked a risk stratification approach. Therefore, in this study, K-Means clustering algorithm was incorporated to stratify the risk of early PPD, ensuring the clinical practicability of the model. In addition, to facilitate clinical application, we developed an online risk calculator to enable accurate and rapid in-hospital assessments of early PPD. This study longitudinally investigated the risk factors for early PPD in a large cohort of Chinese women, and the evaluation indexes of the optimal model were all favorable, confirming its ability to accurately predict the risk of early PPD.

## Limitations

This study had two main limitations. Firstly, the sample data were primarily from China, and further data will be continuously collected for validation through the online web calculator developed in this study. Secondly, while this study provided an early prediction of the risk of PPD and identified key risk factors, future research will focus on refining intervention programs for these key risk factors. This will help provide a reliable foundation for early intervention strategies for PPD.

## Conclusion

In summary, this study identified the risk factors for early PPD based on the social ecosystem theory and constructed a risk prediction model using 10 ML algorithms. The study also performed probability stratification into low, medium, and high-risk categories through K-Means clustering. The model was interpreted using SHAP values, and an online web calculator was developed for practical use. This study provides valuable insights for future research directions and demonstrates the high clinical applicability of the model. It enables healthcare providers to assess the risk of PPD early, offering crucial guidance for its early prevention and intervention.

## Data Availability

The raw data supporting the conclusions of this article will be made available by the authors, without undue reservation.

## References

[1] American Psychiatric Association. (2013). Diagnostic and Statistical Manual of Mental Disorders, Fifth Edition (DSM-5). Washington: American Psychiatric Publishing.

[2] Meltzer-Brody S Howard LM Bergink V Vigod S Jones I Munk-Olsen T. Postpartum psychiatric disorders. Nat Rev Dis Primers. (2018) 4:18022. doi: 10.1038/nrdp.2018.22, PMID: 29695824

[3] Bianciardi E Vito C Betrò S de Stefano A Siracusano A Niolu C. The anxious aspects of insecure attachment styles are associated with depression either in pregnancy or in the postpartum period. Ann General Psychiatry. (2020) 19:51. doi: 10.1186/s12991-020-00301-7, PMID: 32944057 PMC7488240

[4] Santos Junior HP Rosa Gualda DM de Fátima Araújo Silveira M Hall WA. Postpartum depression: the (in) experience of Brazilian primary healthcare professionals. J Adv Nurs. (2013) 69:1248–58. doi: 10.1111/j.1365-2648.2012.06112.x, PMID: 22882356

[5] Galea LAM Frokjaer VG. Perinatal depression: embracing variability toward better treatment and outcomes. Neuron. (2019) 102:13–6. doi: 10.1016/j.neuron.2019.02.023, PMID: 30946818

[6] Wisner KL Chambers C Sit DKY. Postpartum depression: a major public health problem. JAMA. (2006) 296:2616–8. doi: 10.1001/jama.296.21.2616, PMID: 17148727

[7] Murray L Fiori-Cowley A Hooper R Cooper P. The impact of postnatal depression and associated adversity on early mother-infant interactions and later infant outcome. Child Dev. (1996) 67:2512–26. doi: 10.2307/1131637, PMID: 9022253

[8] Stein A Pearson RM Goodman SH Rapa E Rahman A McCallum M. Effects of perinatal mental disorders on the fetus and child. Lancet (London, England). (2014) 384:1800–19. doi: 10.1016/S0140-6736(14)61277-0, PMID: 25455250

[9] Pope CJ Mazmanian D. Breastfeeding and postpartum depression: an overview and methodological recommendations for future research. Depress Res Treat. (2016) 2016:1–9. doi: 10.1155/2016/4765310, PMID: 27148457 PMC4842365

[10] Letourneau NL Dennis C-L Benzies K Duffett-Leger L Stewart M Tryphonopoulos PD. Postpartum depression is a family affair: addressing the impact on mothers, fathers, and children. Issues Ment Health Nurs. (2012) 33:445–57. doi: 10.3109/01612840.2012.673054, PMID: 22757597

[11] Payne JL Maguire J. Pathophysiological mechanisms implicated in postpartum depression. Front Neuroendocrinol. (2019) 52:165–80. doi: 10.1016/j.yfrne.2018.12.001, PMID: 30552910 PMC6370514

[12] Liu X Wang S Wang G. Prevalence and risk factors of postpartum depression in women: a systematic review and Meta-analysis. J Clin Nurs. (2022) 31:2665–77. doi: 10.1111/jocn.16121, PMID: 34750904

[13] Upadhyay RP Chowdhury R Salehi A Sarkar K Singh SK Sinha B. Postpartum depression in India: a systematic review and meta-analysis. Bull World Health Organ. (2017) 95:706–717C. doi: 10.2471/BLT.17.192237, PMID: 29147043 PMC5689195

[14] US Preventive Services Task Force Curry SJ Krist AH Owens DK Barry MJ Caughey AB. Interventions to prevent perinatal depression: US preventive services task force recommendation statement. JAMA. (2019) 321:580–7. doi: 10.1001/jama.2019.0007, PMID: 30747971

[15] Andersson S Bathula DR Iliadis SI Walter M Skalkidou A. Predicting women with depressive symptoms postpartum with machine learning methods. Sci Rep. (2021) 11:7877. doi: 10.1038/s41598-021-86368-y, PMID: 33846362 PMC8041863

[16] Shin D Lee KJ Adeluwa T Hur J. Machine learning-based predictive modeling of postpartum depression. J Clin Med. (2020) 9:2899. doi: 10.3390/jcm9092899, PMID: 32911726 PMC7564708

[17] Qi W Wang Y Li C He K Wang Y Huang S. Predictive models for predicting the risk of maternal postpartum depression: a systematic review and evaluation. J Affect Disord. (2023) 333:107–20. doi: 10.1016/j.jad.2023.04.026, PMID: 37084958

[18] Al’Aref SJ Maliakal G Singh G van Rosendael AR Ma X Xu Z. Machine learning of clinical variables and coronary artery calcium scoring for the prediction of obstructive coronary artery disease on coronary computed tomography angiography: analysis from the CONFIRM registry. Eur Heart J. (2020) 41:359–67. doi: 10.1093/eurheartj/ehz565, PMID: 31513271 PMC7849944

[19] Singh G Al’Aref SJ Van Assen M Kim TS van Rosendael A Kolli KK. Machine learning in cardiac CT: basic concepts and contemporary data. J Cardiovasc Comput Tomogr. (2018) 12:192–201. doi: 10.1016/j.jcct.2018.04.010, PMID: 29754806

[20] McLeroy KR Bibeau D Steckler A Glanz K. An ecological perspective on health promotion programs. Health Educ Q. (1988) 15:351–77. doi: 10.1177/109019818801500401, PMID: 3068205

[21] Potharst ES Veringa-Skiba I van Broekhuizen E Bögels SM. Mindful with your baby for mothers of infants with (parental) stress in a non-clinical setting: a wait-list controlled pilot trial. BMC Pregnancy Childbirth. (2022) 22:298. doi: 10.1186/s12884-022-04640-z, PMID: 35392847 PMC8991950

[22] Wu M. Questionnaire statistical analysis practice: SPSS operation and application. Chongqing: Chongqing University Press (2018).

[23] Rousson V Zumbrunn T. Decision curve analysis revisited: overall net benefit, relationships to ROC curve analysis, and application to case-control studies. BMC Med Inform Decis Mak. (2011) 11:45. doi: 10.1186/1472-6947-11-45, PMID: 21696604 PMC3148204

[24] Takuma Y Shota I Miyatake H Uematsu S Okamoto R Araki Y. Nomograms to predict the disease-free survival and overall survival after radiofrequency ablation for hepatocellular carcinoma. Int Med. (2018) 57:457–68. doi: 10.2169/internalmedicine.9064-17, PMID: 29151504 PMC5849539

[25] Xiao S. The theoretical foundation and research application of the social support rating scale. Clin Psychiatry. (1994):98–100.

[26] Cox JL Holden JM Sagovsky R. Detection of postnatal depression. Development of the 10-item Edinburgh postnatal depression scale. Br J Psychiatry. (1987) 150:782–6. doi: 10.1192/bjp.150.6.782, PMID: 3651732

[27] Shorey S Chee CYI Ng ED Chan YH Tam WWS Chong YS. Prevalence and incidence of postpartum depression among healthy mothers: a systematic review and meta-analysis. J Psychiatr Res. (2018) 104:235–48. doi: 10.1016/j.jpsychires.2018.08.001, PMID: 30114665

[28] Clayborne ZM Colman I Kingsbury M Torvik FA Gustavson K Nilsen W. Prenatal work stress is associated with prenatal and postnatal depression and anxiety: findings from the Norwegian mother, father and child cohort study (MoBa). J Affect Disord. (2022) 298:548–54. doi: 10.1016/j.jad.2021.11.024, PMID: 34774976

[29] Glasser S Barell V Boyko V Ziv A Lusky A Shoham A. Postpartum depression in an Israeli cohort: demographic, psychosocial and medical risk factors. J Psychosom Obstet Gynecol. (2000) 21:99–108. doi: 10.3109/01674820009075615, PMID: 10994182

[30] Walker AL de Rooij SR Dimitrova MV Witteveen AB Verhoeven CJ de Jonge A. Psychosocial and peripartum determinants of postpartum depression: findings from a prospective population-based cohort. The ABCD study. Compr Psychiatry. (2021) 108:152239. doi: 10.1016/j.comppsych.2021.152239, PMID: 33905988

[31] Sobol M Błachnio A Meisner M Szyszkowska J Jankowski KS. Sleep, circadian activity patterns and postpartum depression: a systematic review and meta-analysis of actigraphy studies. J Sleep Res. (2024) 33:e14116. doi: 10.1111/jsr.14116, PMID: 38095248

[32] Park EM Meltzer-Brody S Stickgold R. Poor sleep maintenance and subjective sleep quality are associated with postpartum maternal depression symptom severity. Arch Womens Ment Health. (2013) 16:539–47. doi: 10.1007/s00737-013-0356-9, PMID: 23733081 PMC5308064

[33] Knutson KL Spiegel K Penev P van Cauter E. The metabolic consequences of sleep deprivation. Sleep Med Rev. (2007) 11:163–78. doi: 10.1016/j.smrv.2007.01.002, PMID: 17442599 PMC1991337

[34] Liu Y Croft JB Wheaton AG Perry GS Chapman DP Strine TW. Association between perceived insufficient sleep, frequent mental distress, obesity and chronic diseases among US adults, 2009 behavioral risk factor surveillance system. BMC Public Health. (2013) 13:84. doi: 10.1186/1471-2458-13-84, PMID: 23360346 PMC3562519

[35] Matricciani L Bin YS Lallukka T Kronholm E Wake M Paquet C. Rethinking the sleep-health link. Sleep Health. (2018) 4:339–48. doi: 10.1016/j.sleh.2018.05.004, PMID: 30031526

[36] Shankar A Syamala S Kalidindi S. Insufficient rest or sleep and its relation to cardiovascular disease, diabetes and obesity in a national, multiethnic sample. PLoS One. (2010) 5:e14189. doi: 10.1371/journal.pone.0014189, PMID: 21152066 PMC2995019

[37] Umuziga PM Gishoma D Hynie M Nyirazinyoye L Nsereko E. Predicting postnatal depressive symptoms in a prospective cohort study in Rwanda: the impact of poor maternal social support. Front Glob Womens Health. (2023) 4:1113483. doi: 10.3389/fgwh.2023.1113483, PMID: 37547130 PMC10402918

[38] Phukuta NSJ Omole OB. Erratum: prevalence and risk factors associated with postnatal depression in a south african primary care facility. Afr J Prim Health Care Fam Med. (2021) 13:3179. doi: 10.4102/phcfm.v13i1.3179, PMID: 34931524 PMC8678938

[39] Deng A-W Xiong R-B Jiang T-T Luo YP Chen WZ. Prevalence and risk factors of postpartum depression in a population- based sample of women in Tangxia community, Guangzhou. Asian Pac J Trop Med. (2014) 7:244–9. doi: 10.1016/S1995-7645(14)60030-4, PMID: 24507649

[40] Lau Y Wong DFK. The role of social support in helping Chinese women with perinatal depressive symptoms cope with family conflict. J Obstet Gynecol Neonatal Nurs. (2008) 37:556–71. doi: 10.1111/j.1552-6909.2008.00273.x, PMID: 18811775

[41] Gebregziabher NK Netsereab TB Fessaha YG Alaza FA Ghebrehiwet NK Sium AH. Prevalence and associated factors of postpartum depression among postpartum mothers in central region, Eritrea: a health facility based survey. BMC Public Health. (2020) 20:1614. doi: 10.1186/s12889-020-09676-4, PMID: 33109137 PMC7590801

[42] Hanach N Radwan H Fakhry R Dennis CL issa WB Faris MAIE. Prevalence and risk factors of postpartum depression among women living in the United Arab Emirates. Soc Psychiatry Psychiatr Epidemiol. (2023) 58:395–407. doi: 10.1007/s00127-022-02372-1, PMID: 36239744 PMC9971080

[43] Brown A Rance J Warren L. Body image concerns during pregnancy are associated with a shorter breast feeding duration. Midwifery. (2015) 31:80–9. doi: 10.1016/j.midw.2014.06.003, PMID: 25151278

[44] Hodgkinson EL Smith DM Wittkowski A. Women’s experiences of their pregnancy and postpartum body image: a systematic review and meta-synthesis. BMC Pregnancy Childbirth. (2014) 14:330. doi: 10.1186/1471-2393-14-330, PMID: 25248649 PMC4261580

[45] Mariam KA Srinivasan K. Antenatal psychological distress and postnatal depression: a prospective study from an urban clinic. Asian J Psychiatr. (2009) 2:71–3. doi: 10.1016/j.ajp.2009.04.002, PMID: 23051032

[46] Yang S-T Yang S-Q Duan K-M Tang YZ Ping AQ Bai ZH. The development and application of a prediction model for postpartum depression: optimizing risk assessment and prevention in the clinic. J Affect Disord. (2022) 296:434–42. doi: 10.1016/j.jad.2021.09.099, PMID: 34606808

[47] Hochman E Feldman B Weizman A Krivoy A Gur S Barzilay E. Development and validation of a machine learning-based postpartum depression prediction model: a nationwide cohort study. Depress Anxiety. (2021) 38:400–11. doi: 10.1002/da.23123, PMID: 33615617

[48] Zhang Y Wang S Hermann A Joly R Pathak J. Development and validation of a machine learning algorithm for predicting the risk of postpartum depression among pregnant women. J Affect Disord. (2021) 279:1–8. doi: 10.1016/j.jad.2020.09.113, PMID: 33035748 PMC7738412

