# Supplementary material for: Development and validation of an interpretable machine learning model and online web-based calculator based on social-ecosystem theory for early prediction of postpartum depression: a longitudinal study
Source: Front Public Health. 2025 Oct 17;13:1685305. doi: 10.3389/fpubh.2025.1685305 (PMC12575091; doi:10.3389/fpubh.2025.1685305)
Supplement: Supplementary file 1 [file Supplementary_file_1.docx]

The Early Risk Factors for PPD Questionnaire

1.What’s Your Age?

2.Your Husband’s Age?

3.What's Your Educational Background?

Primary school and below

Junior high school or secondary school

High school or college

Undergraduate

Master's degree or above

4.What’s Your Husband’s Educational Background?

Primary school and below

Junior high school or secondary school

High school or college

Undergraduate

Master's degree or above

5.What’s Your Marital Status?

Unmarried

First marriage

Remarriage

Get divorced

Widowed spouse

6.Maternal Pre-Pregnancy Average Monthly Income

No income

< 3,000 CNY

3,000-5,999 CNY

6,000-9,999 CNY

10,000-20,000 CNY

> 20,000 CNY

7.Maternity Leave Duration

< 1 month

1-3 months

4-6 months

7-8 months

9-12 months

More than 1 year

8.Work Stress During Maternity Leave

No

Yes

9.Maternity Insurance

No

Yes

10.Stable Income During Maternity Leave

No

Yes

11.Husband’s Average Monthly Income

No income

< 3,000 CNY

3,000-5,999 CNY

6,000-9,999 CNY

10,000-20,000 CNY

> 20,000 CNY

12.Husband’s Care Leave

< 1 week

1-2 weeks

3-4 weeks

4-8 weeks

More than 8 weeks

13.Can the Husband Provide Sufficient Care?

Yes

No

14.Postpartum Recovery Place

Own home

Postpartum care center

Others

15.Maternal Evaluation of Living Place

Spacious and comfortable

Average

Poor conditions

Very poor conditions

16.Are Community Neighborly Relations Harmonious?

No

Yes

17.Is Medical Care Convenient at the Place of Residence?

No

Yes

18.Maternal Evaluation of Designated Maternity Healthcare Facility

Excellent

Good

Average

Poor

Very poor

19.Household Members Living Together (Select all that apply)

Husband and Children

Husband’s Parents

Own Parents

Husband’s Siblings

Wife’s Siblings

Maternity Matron or Nanny

Others

20.Maternal Evaluation of Family Relationships

Excellent

Good

Average

Poor

Very poor

21.Relationship with Husband

Excellent

Good

Average

Poor

Very poor

22.Relationship with Parents-in-law

Excellent

Good

Average

Poor

Very poor

23.Relationship with Parents

Excellent

Good

Average

Poor

Very poor

24.Was This Pregnancy Planned?

No

Yes

25.Parity

First birth

Second birth

Third birth

More than three births

26.Preferred Baby Gender During Pregnancy

Boy

Girl

One boy, one girl

Two girls

Two boys

Multiple births

No preference

27.Maternal Attitude Toward Actual Baby Gender

Very satisfied

No preference

Not satisfied

28.Do Family Members Have Gender Bias Toward the Baby?

No

Yes

29.Has the Mother Experienced Domestic Violence?

No

Yes

30.Can the Family Provide Sufficient Daily Life and Care Support?

No

Yes

31.Can the Family Provide Sufficient Infant Care Support?

No

Yes

32.Does the Mother Have Someone to Confide in When Conflicts Arise with Family Members?

No

Yes

33.Was Folic Acid Supplemented Regularly During Pregnancy?

No

Regularly

Irregularly

34.Regular prenatal care

No

Yes

35.Pre-Pregnancy Height (m) and Weight (kg)

36.Pre-Delivery Height (m) and Weight (kg)

37.Did Pregnancy Weight Gain Cause Distress?

No

Yes

38.Whether the following conditions occurred during pregnancy? (Select all that apply)

Gestational Diabetes

Gestational Hypertension

Hyperthyroidism

Hypothyroidism

Threatened Miscarriage

Threatened Preterm Labor

Other Conditions

39.Weight of the newborn

40.Whether the newborn is currently ill?

41.Current Baby Feeding Method

Breastfeeding

Mixed feeding

Formula feeding

42.Baby’s Characteristics

Well-behaved

Easily cries

Difficult to feed

43.Current Maternal Sleep Condition

Excellent

Good

Average

Poor

Very poor

44.Maternal Confidence in Newborn Care

Very confident

Fairly confident

Average confidence

No confidence

45.Does Postpartum Diet Meet Personal Preferences?

No

Yes

46.Was the Mother Forced to Eat Due to Breastfeeding?

No

Yes

47.Personality Type (Select all that apply)

Extroverted

Introverted

Mixed

Stable

Unstable

48.Time from Regular Contractions to Delivery

<4 hours

4-6 hours

>6 hours

49.Feelings During Delivery (Select all that apply)

Excited

Nervous

Painful

Happy

Other

50.Family history of mental illness

No

Yes

51.Mode of delivery

Vaginal delivery

Cesarean delivery

Painless delivery

52.Labor Pain Relief Method

None

Epidural analgesia

Intravenous analgesia

Non-pharmacological pain relief

General anesthesia

Other

53.Level of Social Support

(The Social Support Rating Scale (SSRS) developed by Xiao Shuiyuan (1994) was used to evaluate this part. The scale had 10 items divided into 3 dimensions: subjective support (items 1, 3, 4, 5), objective material support (items 2, 6, 7), and utilization of support (items 8, 9, 10). See the Methods section for details.)
