# Supplementary material for: Development and validation of an interpretable machine learning model and online web-based calculator based on social-ecosystem theory for early prediction of postpartum depression: a longitudinal study
Source: Front Public Health. 2025 Oct 17;13:1685305. doi: 10.3389/fpubh.2025.1685305 (PMC12575091; doi:10.3389/fpubh.2025.1685305)
Supplement: Supplementary file 2 [file Table_1.docx]

***Appendices***

**Table S1** Univariate analysis of risk factors for early postpartum depression.

**Table S2** Assignments of independent variables.

**Table S3** Diagnosis of collinearity.

**Table S1**

Univariate analysis of risk factors for early postpartum depression.

| **Variables** | **PPD**  **(n=201)** | **non-PPD**  **(n=362)** | ***P-***  **value** | |
| --- | --- | --- | --- | --- |
| X1-Maternal age,years | 32.38$\pm$4.78 | 31.83$\pm$4.91 | 0.199 |  |
| X2-Husband’s age,years | 34.73$\pm$7.21 | 33.76$\pm$6.40 | 0.102 |  |
| X3-Maternal education |  |  | 0.167 |  |
| Primary school and below  Junior high school or secondary school  High school or college  Undergraduate  Master’s degree or above | 0(0.0)  12(6.0)  65(32.3)  86(42.8)  38(18.9) | 0(0.0)  35(9.7)  131(36.2)  125(34.5)  71(19.6) |  |  |
| X4-Husband’s education |  |  | 0.683 |  |
| Primary school and below  Junior high school or secondary school  High school or college  Undergraduate  Master's degree or above | 2(1.0)  10(5.0)  63(31.3)  91(45.3)  35(17.4) | 0(0.0)  41(11.3)  96(26.5)  160(44.2)  65(18.0) |  |  |
| X5-Marital status |  |  | 0.316 |  |
| Unmarried  First marriage  Remarriage  Get divorced  Widowed spouse | 4(2.0)  192(95.5)  5(2.5)  0(0.0)  0(0.0) | 3(0.8)  345(95.3)  14(3.9)  0(0.0)  0(0.0) |  |  |
| X6-Maternal Pre-Pregnancy Average Monthly Income |  |  | 0.801 |  |
| No income  < 3,000 CNY  3,000-5,999 CNY  6,000-9,999 CNY  10,000-20,000 CNY  > 20,000 CNY | 15(7.5)  8(4.0)  58(28.9)  89(44.3)  28(13.9)  3(1.5) | 34(9.4)  11(3.0)  110(30.4)  153(42.3)  44(12.2)  10(2.8) |  |  |
| X7-Maternity Leave Duration |  |  | 0.494 |  |
| < 1 month  1-3 months  4-6 months  7-8 months  9-12 months  More than 1 year | 5(2.5)  21(10.4)  143(71.1)  4(2.0)  0  28(13.9) | 13(3.6)  23(6.4)  267(73.8)  10(2.8)  2(0.6)  47(13.0) |  |  |
| X8-Work Stress During Maternity Leave |  |  | <0.001 |  |
| No  Yes | 69(34.3)  132(65.7) | 181(50.0)  181(50.0) |  |  |
| X9-Maternity Insurance |  |  | 0.058 |  |
| No  Yes | 22(10.9)  179(89.1) | 61(16.9)  301(83.1) |  |  |
| X10-Stable Income During Maternity Leave |  |  | 0.528 |  |
| No  Yes | 56(27.9)  145(72.1) | 92(25.4)  270(74.6) |  |  |
| X11-Husband’s Average Monthly Income |  |  | 0.655 |  |
| No income  < 3,000 CNY  3,000-5,999 CNY  6,000-9,999 CNY  10,000-20,000 CNY  ＞20,000 CNY | 0(0.0)  1(0.5)  32(15.9)  83(41.3)  64(31.8)  21(10.4) | 2(0.6)  6(1.7)  71(19.6)  138(38.1)  109(30.1)  36(9.9) |  |  |
| X12-Husband’s Care Leave |  |  | 0.121 |  |
| < 1 week  1-2 weeks  3-4 weeks  4-8 weeks  More than 8 weeks | 40(19.9)  113(56.2)  29(14.4)  3(1.5)  16(8.0) | 60(16.6)  208(57.5)  37(10.2)  15(4.1)  42(11.6) |  |  |
| X13-Can the Husband Provide Sufficient Care? |  |  | <0.001 |  |
| Yes  No | 154(76.6)  47(23.4) | 329(90.9)  33(9.1) |  |  |
| X14-Postpartum Recovery Place |  |  | 0.029 |  |
| Own home  Postpartum care center  Others | 159(79.1)  21(10.4)  21(10.4) | 317(87.6)  23(6.4)  22(6.1) |  |  |
| X15-Maternal Evaluation of Living Place |  |  | 0.003 |  |
| Spacious and comfortable  Average  Poor conditions  Very poor conditions | 143(71.1)  57(28.4)  1(0.5)  0(0.0) | 297(82.0)  64(17.7)  1(0.3)  0(0.0) |  |  |
| X16-Are Community Neighborly Relations Harmonious? |  |  | 0.270 |  |
| No  Yes | 1(0.5)  200(99.5) | 7(1.9)  355(98.1) |  |  |
| X17-Is Medical Care Convenient at the Place of Residence? |  |  | 0.433 |  |
| No  Yes | 18(9.0)  183(91.0) | 40(11.0)  322(89.0) |  |  |
| X18-Maternal Evaluation of Designated Maternity Healthcare Facility |  |  | 0.783 |  |
| Excellent  Good  Average  Poor  Very poor | 151(75.1)  39(19.4)  11(5.5)  0(0.0)  0(0.0) | 276(76.2)  66(18.2)  20(5.5)  0(0.0)  0(0.0) |  |  |
| X19-Household Members Living Together  X19.1 Husband and Children |  |  | 0.281 |  |
| No  Yes | 30(14.9)  171(85.1) | 67(18.5)  295(81.5) |  |  |
| X19.2 Husband’s Parents |  |  | 0.250 |  |
| No  Yes | 131(65.2)  70(34.8) | 253(69.9)  109(30.1) |  |  |
| X19.3 Own Parents |  |  | 0.448 |  |
| No  Yes | 162(80.6)  39(19.4) | 301(83.1)  61(16.9) |  |  |
| X19.4 Husband’s Siblings |  |  | 0.940 |  |
| No  Yes | 199(99.0)  2(1.0) | 360(99.4)  2(0.6) |  |  |
| X19.5 Wife’s Siblings |  |  | 0.540 |  |
| No  Yes | 201(100.0)  0(0.0) | 360(99.4)  2(0.6) |  |  |
| X19.6 Maternity Matron or Nanny |  |  | 0.287 |  |
| No  Yes | 177(88.1)  24(11.9) | 329(90.9)  33(9.1) |  |  |
| X19.7 Others |  |  | 0.067 |  |
| No  Yes | 199(99.0)  2(1.0) | 349(96.4)  13(3.6) |  |  |
| X20-Maternal Evaluation of Family Relationships |  |  | <0.001 |  |
| Excellent  Good  Average  Poor  Very poor | 114(56.7)  72(35.8)  11(5.5)  4(2.0)  0(0.0) | 272(75.1)  70(19.3)  16(4.4)  4(1.1)  0(0.0) |  |  |
| X21-Relationship with Husband |  |  | <0.001 |  |
| Excellent  Good  Average  Poor  Very poor | 138(68.7)  60(29.9)  3(1.5)  0(0.0)  0(0.0) | 304(84.0)  52(14.4)  6(1.7)  0(0.0)  0(0.0) |  |  |
| X22-Relationship with Parents-in-law |  |  | <0.001 |  |
| Excellent  Good  Average  Poor  Very poor | 93(46.3)  78(38.8)  30(14.9)  0(0.0)  0(0.0) | 256(70.7)  81(22.4)  24(6.6)  1(0.3)  0(0.0) |  |  |
| X23-Relationship with Parents |  |  | <0.001 |  |
| Excellent  Good  Average  Poor  Very poor | 125(62.2)  66(32.8)  7(3.5)  0(0.0)  3(1.5) | 287(79.3)  65(18.0)  7(1.9)  0(0.0)  3(0.8) |  |  |
| X24-Was This Pregnancy Planned? |  |  | 0.558 |  |
| No  Yes | 18(9.0)  183(91.0) | 38(10.5)  324(89.5) |  |  |
| X25-Parity |  |  | 0.852 |  |
| First birth  Second birth  Third birth  More than three births | 149(74.1)  47(23.4)  5(2.5)  0(0.0) | 259(71.5)  91(25.1)  10(2.8)  2(0.6) |  |  |
| X26-Preferred Baby Gender During Pregnancy |  |  | 0.957 |  |
| Boy  Girl  One boy, one girl  Two girls  Two boys  Multiple births  No preference | 26(12.9)  40(19.9)  15(7.5)  0(0.0)  0(0.0)  1(0.5)  119(59.2) | 46(12.7)  69(19.1)  23(6.4)  0(0.0)  0(0.0)  1(0.3)  223(61.6) |  |  |
| X27-Maternal Attitude Toward Actual Baby Gender |  |  | 0.059 |  |
| Very satisfied  No preference  Not satisfied | 133(66.2)  1(0.5)  67(33.3) | 266(73.5)  4(1.1)  92(25.4) |  |  |
| X28-Do Family Members Have Gender Bias Toward the Baby? |  |  | 0.367 |  |
| No  Yes | 5(2.5)  196(97.5) | 4(1.1)  358(98.9) |  |  |
| X29-Has the Mother Experienced Domestic Violence? |  |  | 1.000 |  |
| No  Yes | 199(99.0)  2(1.0) | 357(98.6)  5(1.4) |  |  |
| X30-Can the Family Provide Sufficient Daily Life and Care Support? |  |  | 0.318 |  |
| No  Yes | 6(3.0)  195(97.0) | 5(1.4)  357(98.6) |  |  |
| X31-Can the Family Provide Sufficient Infant Care Support? |  |  | 0.092 |  |
| No  Yes | 12(6.0)  189(94.0) | 11(3.0)  351(97.0) |  |  |
| X32-Does the Mother Have Someone to Confide in When Conflicts Arise with Family Members? |  |  | 0.788 |  |
| No  Yes | 22(10.9)  179(89.1) | 37(10.2)  325(89.8) |  |  |
| X33-Was Folic Acid Supplemented Regularly During Pregnancy? |  |  | 0.716 |  |
| No  Regularly  Irregularly | 95(47.3)  82(40.8)  24(11.9) | 159(43.9)  160(44.2)  43(11.9) |  |  |
| X34-Regular prenatal care |  |  | 0.786 |  |
| No  Yes | 3(1.5)  198(98.5) | 8(2.2)  354(97.8) |  |  |
| X35-Pre-Pregnancy BMI | 23.93±6.11 | 24.15±6.55 | 0.697 |  |
| X36-Pre-Delivery BMI | 29.43±7.29 | 29.51±7.62 | 0.907 |  |
| X37-Did Pregnancy Weight Gain Cause Distress? |  |  | <0.001 |  |
| No  Yes | 89(44.3)  112(55.7) | 300(82.9)  62(17.1) |  |  |
| X38-Whether the following conditions occurred during pregnancy |  |  |  |  |
| X38.1 Gestational Diabetes |  |  | 0.924 |  |
| No  Yes | 157(78.1)  44(21.9) | 284(78.5)  78(21.5) |  |  |
| X38.2 Gestational Hypertension |  |  | 0.331 |  |
| No  Yes | 189(94.0)  12(6.0) | 347(95.9)  15(4.1) |  |  |
| X38.3 Hyperthyroidism |  |  | 0.168 |  |
| No  Yes | 200(99.5)  1(0.5) | 353(97.5)  9(2.5) |  |  |
| X38.4 Hypothyroidism |  |  | 0.394 |  |
| No  Yes | 185(92.0)  16(8.0) | 340(93.9)  22(6.1) |  |  |
| X38.5 Threatened Miscarriage |  |  | 0.019 |  |
| No  Yes | 173(86.1)  28(13.9) | 334(92.3)  28(7.7) |  |  |
| X38.6 Threatened Preterm Labor |  |  | 0.255 |  |
| No  Yes | 183(91)  18(9.0) | 339(93.6)  23(6.4) |  |  |
| X38.7 Other Conditions |  |  | 0.505 |  |
| No  Yes | 191(95.0)  10(5.0) | 339(93.6)  23(6.4) |  |  |
| X39-Low birth weight |  |  | 0.472 |  |
| No  Yes | 185(92.0)  16(8.0) | 339(93.6)  23(6.4) |  |  |
| X40-Is the Newborn Healthy? |  |  | 0.177 |  |
| No  Yes | 7(3.5)  194(96.5) | 5(1.4)  357(98.6) |  |  |
| X41-Current Baby Feeding Method |  |  | 0.908 |  |
| Breastfeeding  Mixed feeding  Formula feeding | 76(37.8)  105(52.2)  20(10.0) | 132(36.5)  196(54.1)  34(9.4) |  |  |
| X42-Baby’s Characteristics |  |  | 0.896 |  |
| Well-behaved  Easily cries  Difficult to feed | 158(78.6)  38(18.9)  5(2.5) | 284(8.8)  5(2.5)  7(1.9) |  |  |
| X43-Current Maternal Sleep Condition |  |  | <0.001 |  |
| Excellent  Good  Average  Poor  Very poor | 20(10.0)  31(15.4)  40(19.9)  83(41.3)  0(0.0) | 86(23.8)  95(26.2)  59(16.3)  104(28.7)  0(0.0) |  |  |
| X44-Maternal Confidence in Newborn Care |  |  | 0.252 |  |
| Very confident  Fairly confident  Average confidence  No confidence | 95(47.3)  76(37.8)  26(12.9)  4(2.0) | 183(50.6)  142(39.2)  32(8.8)  5(1.4) |  |  |
| X45-Does Postpartum Diet Meet Personal Preferences? |  |  | 0.351 |  |
| No  Yes | 30(14.9)  171(85.1) | 44(12.2)  318(87.8) |  |  |
| X46-Was the Mother Forced to Eat Due to Breastfeeding? |  |  | 0.001 |  |
| No  Yes | 143(71.1)  58(28.9) | 300(82.9)  62(17.1) |  |  |
| X47-Personality Type  X47.1 Extroverted |  |  | 0.366 |  |
| No  Yes | 131(65.2)  70(34.8) | 222(61.3)  140(38.7) |  |  |
| X47.2 Introverted |  |  | 0.022 |  |
| No  Yes | 153(76.1)  48(23.9) | 304(84.0)  58(16.0) |  |  |
| X47.3 Mixed |  |  | 0.577 |  |
| No  Yes | 119(59.2)  82(40.8) | 223(61.6)  139(38.4) |  |  |
| X47.4 Stable |  |  | 0.264 |  |
| No  Yes | 129(64.2)  72(35.8) | 215(59.4)  147(40.6) |  |  |
| X47.5 Unstable |  |  | 0.808 |  |
| No  Yes | 182(90.5)  19(9.5) | 330(91.2)  32(8.8) |  |  |
| X48-Time from Regular Contractions to Delivery |  |  | 0.145 |  |
| < 4 hours  4-6 hours  > 6 hours | 85(42.3)  62(30.8)  54(26.9) | 182(50.3)  104(28.7)  76(21.0) |  |  |
| X49-Feelings During Delivery  X49.1 Excited |  |  | 0.031 |  |
| No  Yes | 181(90.0)  20(10.0) | 302(83.4)  60(16.6) |  |  |
| X49.2 Nervous |  |  | 0.323 |  |
| No  Yes | 78(38.8)  123(61.2) | 156(43.1)  206(56.9) |  |  |
| X49.3 Painful |  |  | 0.069 |  |
| No  Yes | 79(39.3)  122(60.7) | 171(47.2)  191(52.8) |  |  |
| X49.4 Happy |  |  | 0.107 |  |
| No  Yes | 167(83.1)  34(16.9) | 280(77.3)  82(22.7) |  |  |
| X49.5 Other |  |  | 0.472 |  |
| No  Yes | 190(94.5)  11(5.5) | 347(95.9)  15(4.1) |  |  |
| X50-Family history of mental illness |  |  | 0.225 |  |
| No  Yes | 194(96.5)  7(3.5) | 341(94.2)  21(5.8) |  |  |
| X51-Mode of delivery |  |  | 0.762 |  |
| Vaginal delivery  Cesarean delivery  Painless delivery | 63(31.3)  133(66.2)  5(2.5) | 123(34.0)  232(64.1)  7(1.9) |  |  |
| X52-Labor Pain Relief Method |  |  | 0.345 |  |
| None  Epidural analgesia  Intravenous analgesia  Non-pharmacological pain relief  General anesthesia  Other | 38(18.9)  128(63.7)  3(1.5)  0(0.0)  24(11.9)  8(4.0) | 61(16.9)  248(68.5)  12(3.3)  0(0.0)  31(8.6)  10(2.8) |  |  |
| X53-Level of Social Support |  |  | <0.001 |  |
| High  Average  Low | 63(31.3)  87(43.3)  51(25.4) | 165(45.6)  167(46.1)  30(8.3) |  |  |

**Table S2**

Assignments of independent variables.

| **Independent variables** | **Assignment** |
| --- | --- |
| X8-Work Stress During Maternity Leave | No=0,Yes=1 |
| X13-Can the Husband Provide Sufficient Care? | Yes=1,No=2 |
| X14-Postpartum Recovery Place | Own home(Z1=0,Z2=0);Postpartum care center(Z1=1,Z2=0);Others(Z1=0,Z2=1) |
| X15-Maternal Evaluation of Living Place | Spacious and comfortable=1;Average=2;Poor conditions=3;Very poor conditions=4 |
| X20-Maternal Evaluation of Family Relationships | Excellent=1;Good=2;Average=3;Poor=4;Very poor=5 |
| X21-Relationship with Husband | Excellent=1;Good=2;Average=3;Poor=4;Very poor=5 |
| X22-Relationship with Parents-in-law | Excellent=1;Good=2;Average=3;Poor=4;Very poor=5 |
| X23-Relationship with Parents | Excellent=1;Good=2;Average=3;Poor=4;Very poor=5 |
| X37-Did Pregnancy Weight Gain Cause Distress? | No=0,Yes=1 |
| X38.6-Whether the following conditions occurred during pregnancy-Threatened Preterm Labor | No=0,Yes=1 |
| X43-Current Maternal Sleep Condition | Excellent=1;Good=2;Average=3;Poor=4;Very poor=5 |
| X46-Was the Mother Forced to Eat Due to Breastfeeding? | No=0,Yes=1 |
| X47.2-Personality Type-Introverted | No=0,Yes=1 |
| X49.1-Feelings During Delivery-Excited | No=0,Yes=1 |
| X53-Level of Social Support | High=1;Average=2;Low=3 |

**Table S3**

Diagnosis of collinearity.

| **Independent variables** | **VIF** |
| --- | --- |
| X8-Work Stress During Maternity Leave | 1.031 |
| X13-Can the Husband Provide Sufficient Care? | 1.206 |
| X14-Postpartum Recovery Place | 1.002 |
| X22-Relationship with Parents-in-law | 1.005 |
| X37-Did Pregnancy Weight Gain Cause Distress? | 1.005 |
| X43-Current Maternal Sleep Condition | 1.009 |
| X53-Level of Social Support | 1.226 |
